# Patient-Level Classification of Rotator Cuff Tears on Shoulder MRI Using an Explainable Vision Transformer Framework

**DOI:** 10.3390/jcm15030928

**Published:** 2026-01-23

**Authors:** Murat Aşçı, Sergen Aşık, Ahmet Yazıcı, İrfan Okumuşer

**Affiliations:** 1 Department of Orthopedics and Traumatology, Faculty of Medicine, Bilecik Şeyh Edebali University, Bilecik 11230, Türkiye; 2 Department of Software Engineering, Faculty of Engineering and Architecture, Eskişehir Osmangazi University, Eskişehir 26040, Türkiye; sergen.asik@ogu.edu.tr; 3 Center of Intelligent Systems Applications Research, Eskişehir Osmangazi University, Eskişehir 26040, Türkiye; 4 Department of Computer Engineering, Faculty of Engineering and Architecture, Eskişehir Osmangazi University, Eskişehir 26040, Türkiye; ayazici@ogu.edu.tr; 5 Department of Radiology, Yunus Emre State Hospital, Ministry of Health, Eskişehir 26190, Türkiye; irfanokumuser@hotmail.com

**Keywords:** rotator cuff tear, magnetic resonance imaging, vision transformer, deep learning, multiple instance learning, explainable ai, medical image analysis, automated diagnosis

## Abstract

**Background/Objectives**: Diagnosing Rotator Cuff Tears (RCTs) via Magnetic Resonance Imaging (MRI) is clinically challenging due to complex 3D anatomy and significant interobserver variability. Traditional slice-centric Convolutional Neural Networks (CNNs) often fail to capture the necessary volumetric context for accurate grading. This study aims to develop and validate the Patient-Aware Vision Transformer (Pa-ViT), an explainable deep-learning framework designed for the automated, patient-level classification of RCTs (Normal, Partial-Thickness, and Full-Thickness). **Methods**: A large-scale retrospective dataset comprising 2447 T2-weighted coronal shoulder MRI examinations was utilized. The proposed Pa-ViT framework employs a Vision Transformer (ViT-Base) backbone within a Weakly-Supervised Multiple Instance Learning (MIL) paradigm to aggregate slice-level semantic features into a unified patient diagnosis. The model was trained using a weighted cross-entropy loss to address class imbalance and was benchmarked against widely used CNN architectures and traditional machine-learning classifiers. **Results**: The Pa-ViT model achieved a high overall accuracy of 91% and a macro-averaged F1-score of 0.91, significantly outperforming the standard VGG-16 baseline (87%). Notably, the model demonstrated superior discriminative power for the challenging Partial-Thickness Tear class (ROC AUC: 0.903). Furthermore, Attention Rollout visualizations confirmed the model’s reliance on genuine anatomical features, such as the supraspinatus footprint, rather than artifacts. **Conclusions**: By effectively modeling long-range dependencies, the Pa-ViT framework provides a robust alternative to traditional CNNs. It offers a clinically viable, explainable decision support tool that enhances diagnostic sensitivity, particularly for subtle partial-thickness tears.

## 1. Introduction

Rotator cuff tears (RCTs) represent one of the most prevalent and disabling musculoskeletal disorders, particularly in aging populations. Epidemiological studies indicate that the prevalence of RCTs increases significantly with age, affecting up to 50% of individuals over the age of 60 [1,2,3]. Among the rotator cuff tendons, the supraspinatus is the most frequently injured due to its unique anatomical position beneath the coracoacromial arch and its susceptibility to both intrinsic degeneration and extrinsic impingement [4,5,6]. Clinically, accurate differentiation among partial-thickness tears, full-thickness tears, and tendinosis is critical, as it dictates the therapeutic pathway—ranging from conservative management and physical therapy to surgical repair [3,7,8]. Furthermore, associated features such as tendon retraction and fatty infiltration of the muscle belly are key prognostic factors for repairability, postoperative healing, and functional outcomes [1,9,10].

Magnetic Resonance Imaging (MRI) is currently the non-invasive gold standard for diagnosing RCTs, offering superior soft-tissue contrast for evaluating tendon integrity and muscle quality [8,11,12,13]. However, shoulder MRI interpretation is complex and subject to significant interobserver variability, particularly when distinguishing high-grade partial tears from small full-thickness tears [2,11,14,15]. While magnetic resonance arthrography (MRA) can increase diagnostic sensitivity, it is nevertheless invasive and not routinely performed for all patients [8,16]. Consequently, there is growing clinical demand for automated, objective, and reproducible diagnostic support tools to assist radiologists in accurately grading the tendon pathology [8,11,13,15].

In recent years, Artificial Intelligence (AI), particularly deep learning (DL), has revolutionized orthopedic imaging [15,17,18]. Early computational approaches relied on machine-learning classifiers (e.g., Support Vector Machines) using handcrafted texture features such as Gray-Level Co-occurrence Matrix (GLCM) or Local Binary Pattern (LBP) [11,19]. However, these methods often lacked generalizability across different MRI scanners. The advent of Convolutional Neural Networks (CNNs) marked a paradigm shift, achieving expert-level performance in tear detection [2,4,15]. Most existing studies have employed 2D CNN architectures (e.g., ResNet, VGG, Xception) that analyze MRI data on a slice-by-slice basis [13,19,20,21,22]. While effective, these 2D slice-centric models have a fundamental limitation: they often fail to capture the 3D volumetric context and the continuity of the tendon across adjacent slices [22,23]. Although some studies have explored 3D CNNs to address this limitation, such models are computationally expensive and often struggle with the “black-box” nature of deep learning, offering limited explainability [1,2,22].

A current debate in the field concerns whether assessing the shoulder as a sequence of images is superior to analyzing 3D volumes directly or treating 2D slices independently [2,22]. This has motivated the exploration of Vision Transformers (ViTs), a novel architecture that uses self-attention mechanisms to model long-range dependencies [4,11]. Unlike CNNs, which are constrained by local receptive fields, Transformers can attend to global anatomical relationships, more closely reflecting how radiologists scroll through MRI stacks to form a patient-level diagnosis [4,22]. Recent hybrid models that combine CNNs for feature extraction with Transformers for sequence modeling have shown promise in other medical imaging domains but remain underexplored for rotator cuff grading [4]. Several recent studies have explicitly modeled medical imaging examinations as slice-level sequences rather than as independent 2D samples. For instance, attention-based Multiple Instance Learning (MIL) frameworks have been used successfully to aggregate slice-level representations into patient-level predictions, demonstrating improved robustness and interpretability in volumetric imaging tasks. In cardiovascular CT angiography, slice-level attention mechanisms have been used to identify diagnostically relevant slices that contribute to patient-level outcomes [24]. Similarly, hierarchical MIL and attention-based aggregation strategies have been shown to capture inter-slice dependencies effectively without requiring explicit slice-level annotations [25]. These approaches highlight the potential clinical value of sequential or instance-level aggregation for volumetric medical imaging.

The primary aim of this study is to develop and validate a patient-level, explainable Vision Transformer framework for automated classification of supraspinatus tendon pathology (Normal, Partial-Thickness Tear, Full-Thickness Tear). We hypothesize that a Transformer-based architecture that treats the MRI stack as a coherent anatomical sequence will outperform traditional slice-based CNNs in diagnostic accuracy. Furthermore, by leveraging the attention mechanism inherent to Transformers, we aim to generate high-resolution, slice-specific attention maps. These visualizations help bridge the explainability gap by enabling clinicians to verify that the model focuses on relevant anatomical structures—such as the tendon footprint—rather than imaging artifacts. Overall, this study emphasizes clinical integration by moving beyond binary detection to provide a graded, explainable, and patient-centric diagnosis.

Although this study utilizes the same dataset originally introduced by Kim et al. [26], our work makes several distinct and significant contributions. First, we reformulate rotator cuff tear classification as a patient-level Multiple Instance Learning (MIL) problem, rather than relying on slice-level predictions. Second, we introduce a Vision Transformer–based architecture that leverages global self-attention to capture long-range anatomical dependencies across slices. Third, we provide model transparency through attention rollout visualizations, enabling anatomical verification of the decision-making process. To the best of our knowledge, this is the first study to combine weakly supervised MIL, Vision Transformers, and explainable attention mechanisms for patient-level rotator cuff tear grading.

## 2. Materials and Methods

The study was conducted in accordance with the Declaration of Helsinki. The research protocol, entitled “Multimodal Diagnosis and Virtual Assistant Use in Magnetic Resonance Imaging for Rotator Cuff Tear Diagnosis,” was reviewed and approved by the Non-Interventional Clinical Research Ethics Committee of Bilecik Şeyh Edebali University (Approval Date: 30 October 2025; Meeting Number: 2025/10; Decision Number: 9).

In this section, we comprehensively delineate the study population, the proposed computational framework—the Patient-Aware Vision Transformer (Pa-ViT)—and the experimental protocols designed for automated, patient-level diagnosis of rotator cuff tears (RCTs) using volumetric magnetic resonance imaging (MRI). The methodology is organized into four principal components: dataset characteristics, mathematical problem formulation, architectural innovations, and the optimization landscape.

### 2.1. Dataset and Study Population

To ensure the clinical relevance and robustness of our evaluation, we utilized a large-scale retrospective dataset comprising 2447 shoulder MRI examinations, as introduced by Kim et al. [26]. The dataset consists exclusively of T2-weighted images, with a particular emphasis on coronal slices, which are widely regarded as the gold standard for evaluating the integrity of the rotator cuff tendons due to the high contrast between hypointense (dark) tendon fibers and hyperintense (bright) fluid signals associated with tears.

Imaging studies were included if they contained complete T2-weighted sequences acquired in oblique coronal, sagittal, and axial planes, which together constitute the standard shoulder MRI protocol for comprehensive rotator cuff assessment. Examinations that did not meet ideal shoulder MRI acquisition criteria—including incomplete imaging planes, missing T2-weighted series, severe motion artifacts, or technically inadequate image quality—were excluded from the study.

#### 2.1.1. Data Characteristics and Class Distribution

As summarized in Table 1, the dataset exhibits a pronounced long-tail distribution that closely reflects real-world clinical prevalence. Normal examinations constitute the majority of cases (66.51%), followed by full-thickness tears (27.06%), while partial-thickness tears represent a smaller but clinically significant minority (6.43%).

#### 2.1.2. Data Partitioning Strategy

To ensure a robust and unbiased evaluation, we employed a strict patient-level split, allocating 1959 examinations to training and 488 to validation/testing. This strategy ensures that imaging slices from the same patient do not appear in both the training and evaluation sets. Dataset partitioning is performed strictly at the patient level: all slices belonging to a given patient are stored under a single patient identifier and are assigned entirely to exactly one split (train/validation/test). This guarantees that no slices from the same patient appear across different splits and prevents optimistic bias due to slice-level leakage. The split is stratified by class, and patient identifiers are shuffled prior to allocation to preserve class proportions across splits, thereby preventing data leakage and enabling a reliable assessment of the model’s generalization capability.

### 2.2. Data Preprocessing and Augmentation

A standard clinical shoulder MRI examination consists of a series of T2-weighted coronal slices that traverse the glenohumeral joint from anterior to posterior. In general, the number of slices per examination ($N_{p}$) can vary across patients depending on body habitus and the MRI scanner’s acquisition protocol. However, in our cohort, $N_{p}$ was constant at 16 slices per patient (median = 16; IQR = 16−16; range = 16−16). We therefore use all available slices for each patient (batch size = 1 during data loading) without subsampling, preserving the full diagnostic context of the scan. Thus, each patient examination can be modeled as a fixed-length sequence of 16 coronal T2-weighted slices, ensuring consistency across the cohort.

To standardize the input for the deep-learning model, the spatial dimensions (H and W) of each slice were normalized to 224 ×224 pixels via bicubic interpolation. The channel dimension C=3 was constructed by replicating the grayscale intensity to accommodate the pre-trained weights of the encoder architecture.

To improve the generalization capability of the model and prevent overfitting, given the class imbalance, we devised a comprehensive data augmentation pipeline applied strictly during the training phase using the torchvision library. The specific transformations included:

- Geometric Transformations: Random rotations (±15°), random affine transformations (scaling range 0.9–1.1, translation 10%), and random horizontal flips (*p* = 0.5).
- Photometric Augmentations: Color Jittering (brightness and contrast adjusted by a factor of 0.1) was employed to simulate the variability in signal-to-noise ratios observed across different MRI scanner manufacturers.
- Normalization: All images were normalized using the standard ImageNet mean ([0.485, 0.456, 0.406]) and standard deviation ([0.229, 0.224, 0.225]).

**Figure 1:**
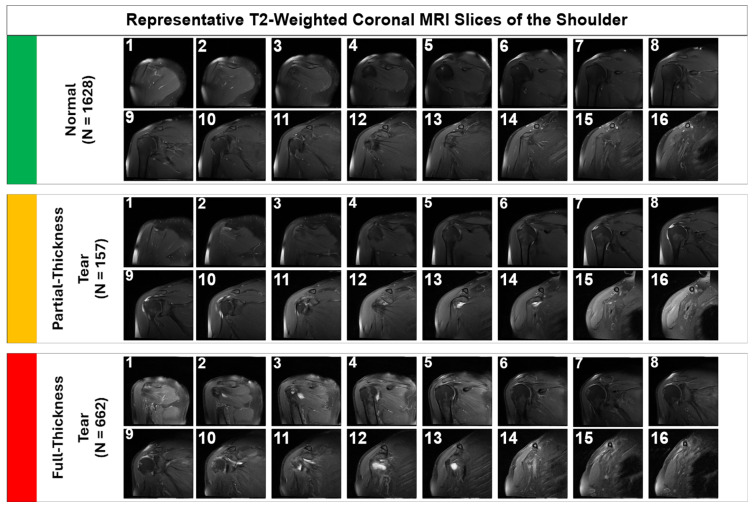
Representative T2-weighted coronal MRI slices from the dataset. (**Top Row**) Normal tendons showing uniform low signal intensity. (**Middle Row**) Partial-thickness tears characterized by focal hyperintensity on the articular side of the tendon. (**Bottom Row**) Full-thickness tears demonstrating complete tendon discontinuity and significant fluid accumulation.

Representative samples illustrating the visual complexity of the diagnostic task are provided in Figure 1. Note the subtle focal hyperintensity of the partial-thickness tear compared to the gross disruption seen in the full-thickness tear.

### 2.3. Problem Formulation: Volumetric Multiple Instance Learning

The diagnosis of RCTs presents a unique set of challenges distinct from standard computer vision tasks: the input is a variable-length sequence of 2D slices rather than a static image; the pathological signal is often subtle and localized to a specific anatomical sub-region (the supraspinatus footprint); and the remaining slices may display healthy anatomy or irrelevant structures. Traditional Deep-Learning approaches that rely on slice-level classification treat each image as an independent sample, a formulation that erroneously discards the critical inter-slice context and necessitates labor-intensive slice-level annotations.

To address these challenges, we rigorously formulate the diagnostic task as a Weakly-Supervised Multiple Instance Learning (MIL) problem. Let X represent the high-dimensional input space of MRI examinations. We define a single patient sample $X_{i}$ not as a static tensor, but as a “bag” of instances composed of a variable sequence of $N_{i}$ slices:

(1) $$X_{i}=\{x_{i,1},x_{i,2},\ldots ,x_{i,N_{i}}\}\;\mathrm{where}\;x_{i,j}\in R^{H\times W\times C},$$

The objective of our framework is to learn a non-linear mapping function $F:X_{i}\to y_{i}$ that predicts the patient-level diagnostic label $y_{i}\in \{\mathrm{Normal},\mathrm{Partial},\mathrm{Full}\}$, essentially treating the patient label as a “weak label” for the entire bag. This formulation forces the algorithm to implicitly learn a relevance function for each instance within the bag, aggregating local evidence to form a global prediction.

### 2.4. Proposed Method: The Patient-Aware Vision Transformer (Pa-ViT) Architecture

**Figure 2:**
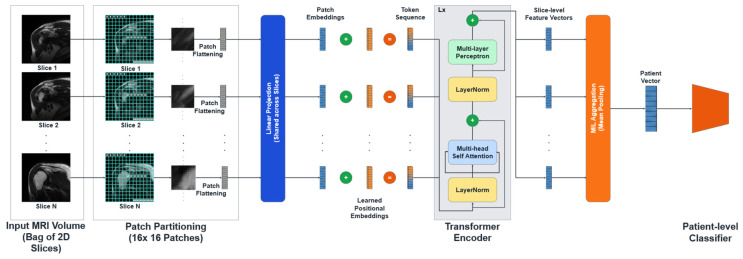
The proposed Patient-Aware Vision Transformer (Pa-ViT) architecture.

Here, the term “patient-aware” refers to a patient-level learning paradigm in which information from all MRI slices belonging to a single patient is aggregated to generate a unified diagnosis; no demographic or clinical metadata were used. While Convolutional Neural Networks (CNNs) have been explored for RCT diagnosis, they process images via local convolution operations, inherently limiting their effective receptive field. However, the radiological signs of a rotator cuff tear often involve subtle semantic relationships between spatially distant anatomical structures. To capture these long-range global dependencies, we propose the Patient-Aware Vision Transformer (Pa-ViT) framework (Figure 2).

The pipeline begins by ingesting a volumetric MRI sequence, which we treat as a bag (set) of 2D slices. Each slice is independently tokenized into patches and processed by a shared Vision Transformer (ViT) encoder to extract high-dimensional semantic embeddings. These slice embeddings are then aggregated using a permutation-invariant mean-pooling operator to form a global patient descriptor.

For a patient (p), we represent the MRI as a set of slices $x_{p,1},\ldots ,x_{p,N_{p}}$. During training and inference: (1) each slice is independently encoded by the backbone to obtain slice embeddings $\left\{z_{p,i}\right\}$; (2) embeddings are aggregated into a patient-level representation via permutation-invariant mean pooling,

(2) $$\bar{z_{p}}=\frac{1}{N_{p}}\sum_{i}z_{p,i};,$$

and (3) a classifier predicts patient-level class probabilities from $\bar{z_{p}}$. This procedure is applied consistently across training, validation, and testing. Moreover, as the data are split at the patient level, there is no overlap of slices between splits.

#### 2.4.1. Slice Encoding and Self-Attention Mechanism

The core feature extraction engine of our framework is a Vision Transformer (ViT-Base) encoder, implemented via the timm library (vit_base_patch16_224). The process begins with Patch Partitioning, where each 2D slice $x_{i,j}$ is tessellated into a grid of non-overlapping patches of resolution P ×P, where P = 16. This operation effectively flattens the 2D image into a sequence of $L=\left(H\cdot W\right)/P^{2}$ vectors, which are then linearly projected into a latent embedding dimension D = 768. Crucially, to allow the model to learn anatomical context—distinguishing the superior aspect of the tendon from the inferior glenoid labrum—we utilize pre-trained 1D positional embeddings.

The sequence of patch embeddings, denoted as $z_{0}$, is processed through a stack L = 12 Transformer layers. The critical computational component within these layers is the Multi-Head Self-Attention (MSA) mechanism. For a given attention head, the input sequence is projected into Query (Q), Key (K), and Value (V) matrices. The attention weights are computed via the scaled dot-product interaction:

(3) $$\mathrm{Attention}\left(Q,K,V\right)=\mathrm{softmax}\left(\frac{QK^{T}}{\sqrt{d_{k}}}\right)V,$$

where $\sqrt{d_{k}}$ is a scaling factor dependent on the embedding dimension ($d_{k}=64$ per head) to ensure numerical stability during gradient backpropagation. This global attention mechanism enables the model to dynamically focus on hyperintense regions indicative of fluid and tears while simultaneously suppressing irrelevant background noise from the deltoid muscle or subcutaneous fat, resulting in a robust slice-level feature vector $f_{i,j}\in R^{D}$.

#### 2.4.2. Permutation-Invariant Patient Aggregation

Following the extraction of slice-level embeddings $\left\{f_{i,1},\ldots ,f_{i,N_{i}}\right\}$, the MIL framework necessitates a differentiable aggregation strategy to synthesize a unified patient-level representation $H_{i}$. We employ a Global Mean Pooling strategy. This choice is theoretically grounded in the requirement for permutation invariance; the diagnostic output of the system should not be dependent on the specific ordering of slices. The aggregation operation is mathematically defined as:

(4) $$H_{i}=\frac{1}{N_{i}}\sum_{j=1}^{N_{i}}f_{i,j},$$

Unlike Max Pooling, which extracts the maximum activation across the volume and risks latching onto a single slice containing a noisy artifact, Mean Pooling aggregates information across the entire volume. This aligns with the radiological reality that true tears are volumetric defects spanning multiple contiguous slices.

#### 2.4.3. Classification Head and Regularization

The aggregated patient vector $H_{i}$ serves as the input to the final classification head. Given the high dimensionality of the feature space relative to the number of unique patients, overfitting is a significant concern. To address this, we designed a custom Multi-Layer Perceptron (MLP) head that incorporates extensive regularization:

- Layer Normalization: Stabilizes the input distribution.
- Linear Projection 1: Projects to a hidden dimension of 512.
- GELU Activation: Utilizing the Gaussian Error Linear Unit for probabilistic non-linearity.
- Dropout (*p* = 0.5): Prevents the co-adaptation of neurons.
- Linear Projection 2: Projects to a dimension of 256, followed by GELU and a second Dropout (*p* = 0.3).
- Final Projection: Maps features to the *C* = 3 diagnostic classes to produce logits.

### 2.5. Optimization Landscape and Implementation Details

This section delineates the technical configuration and optimization strategies employed to ensure the robust performance and reproducibility of the proposed framework.

#### 2.5.1. Loss Function and Class Imbalance Mitigation

A pervasive challenge in medical image analysis is the inherent class imbalance. To ameliorate this, we implemented a Weighted Cross-Entropy Loss combined with Label Smoothing. The class weights $w_{c}$ are strictly calculated using the inverse square root of the class frequency to provide a balanced penalty:

(5) $$w_{c}=\frac{1}{\sqrt{{\mathrm{Frequency}}_{c}}},$$

The final loss function L incorporates these weights along with label smoothing (ϵ= 0.1). Label smoothing replaces the “hard” one-hot target vectors with “soft” targets, preventing the model from becoming over-confident:

(6) $$L=-\sum_{c=1}^{C}w_{c}\cdot \hat{y_{c}}\cdot \log\left(p_{c}\right),$$

#### 2.5.2. Training Protocol and Evaluation Metrics

For optimization, we utilized the AdamW algorithm to decouple weight decay (0.05) from gradient updates. We employed a Cosine Annealing learning rate scheduler and a differential learning rate strategy: the pre-trained backbone was fine-tuned with a conservative learning rate of $5\times 10^{-6}$, while the classification head was trained at $5\times 10^{-5}$. To efficiently manage GPU memory with volumetric 3D data, we utilized Automatic Mixed Precision (AMP)—via torch.amp.GradScaler—and implemented Gradient Accumulation (accumulation steps = 16) to simulate an effective batch size of 16 patients, ensuring stable optimization and convergence.

To comprehensively evaluate the model’s performance, we employed standard classification metrics, including Accuracy, F1-Score (Macro-averaged), Sensitivity, and Specificity. Furthermore, confusion matrices were generated to analyze class-wise performance, particularly for the challenging partial-thickness tear class.

#### 2.5.3. Software, Hardware, and Reproducibility

The entire computational framework was implemented using Python (version 3.8) and the PyTorch deep-learning library (version 2.1.0). The Vision Transformer backbone was implemented using the PyTorch Image Models (timm) library (version 1.0.24), and scikit-learn (version 1.6.1) was used for metric evaluation. Data preprocessing and augmentation were performed using torchvision (version 0.24.0). All experiments were conducted on a Google Colab environment (Google LLC, Mountain View, CA, USA) equipped with an NVIDIA L4 GPU with 24 GB of VRAM. A fixed random seed (seed = 42) was set for all random number generators (NumPy, PyTorch, and Python) to ensure strict reproducibility of the results.

## 3. Results

In this section, we present a comprehensive experimental evaluation of the proposed Pa-ViT framework. The evaluation protocol assesses the model’s performance from three distinct perspectives: quantitative benchmarking against established deep-learning architectures and traditional machine-learning classifiers, granular error analysis to evaluate diagnostic capabilities on clinically challenging classes, and visual validation through Explainable AI (XAI) to ensure the model’s decision-making process aligns with radiological pathology.

### 3.1. Quantitative Benchmarking and Comparative Analysis

To establish the diagnostic efficacy of the Pa-ViT model, we conducted a rigorous comparative analysis. The proposed model was benchmarked against a diverse suite of traditional machine-learning classifiers—including Random Forest, XGBoost, and Support Vector Machines—as well as a strong prior deep-learning baseline for this domain proposed by Kim et al. [26], which utilizes a VGG-16 backbone with Weighted Linear Combination.

The quantitative results, detailed in Table 2, demonstrate that our Vision Transformer-based approach demonstrates improved performance on this dataset. While traditional machine-learning methods struggled to capture the complex features of MRI data, generally yielding accuracies between 52% and 74%, deep-learning approaches showed significantly superior performance.

As presented in Table 2, the Pa-ViT model achieved an Overall Accuracy of 91%, representing a notable improvement over the 87% accuracy reported by the CNN baseline. More importantly, the model achieved a macro-averaged F1-Score of 0.91. This metric is particularly salient in medical imaging, as it confirms that the high accuracy is not merely an artifact of class imbalance (i.e., predicting the majority “Normal” class). The balanced Precision (0.92) and Recall (0.91) indicate that the model effectively minimizes both False Positives (over-diagnosis) and False Negatives (missed diagnosis). This balance is critical for a clinical screening tool, ensuring that healthy patients are not subjected to unnecessary procedures while ensuring pathological cases are not overlooked.

### 3.2. Diagnostic Accuracy and Error Analysis

**Figure 3:**
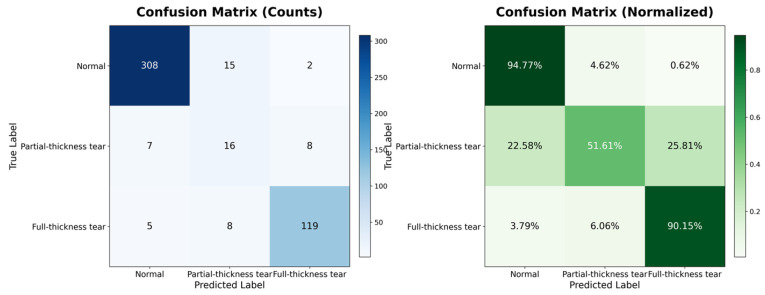
Confusion Matrix of the Pa-ViT model (Left: Absolute Counts, Right: Normalized Percentages). The model exhibits exceptional specificity for Normal cases (94.77%) and high sensitivity for Full-Thickness tears (90.15%).

Beyond global metrics, it is crucial to understand the model’s behavior at a granular level, particularly for classes that are historically difficult to diagnose. To provide insight into the model’s decision-making process, specifically regarding the “Partial-Thickness Tear” class, we examined the Confusion Matrix (Figure 3). This visualization allows us to pinpoint exactly where the model succeeds and where it struggles relative to the ground truth labels.

As illustrated in Figure 3, the model exhibits exceptional specificity for “Normal” examinations, correctly classifying 94.77% of healthy patients. Similarly, for “Full-Thickness Tears,” the sensitivity is remarkably high at 90.15%, indicating the model’s robustness in detecting severe pathology. The most critical insight is derived from the “Partial-Thickness Tear” class. Previous works have struggled significantly with this class due to its subtle morphological features, often reporting accuracies as low as 38%. Our model advances this frontier, correctly identifying 51.61% of partial tears. While misclassifications exist, they are clinically interpretable: 25.81% of partial tears were classified as “Full-Thickness Tears,” likely representing high-grade partial tears that are morphologically similar to full tears. Conversely, only 22.58% were missed as “Normal,” suggesting that the model is sensitive to the presence of pathology even if the severity grading is occasionally overestimated.

**Figure 4:**
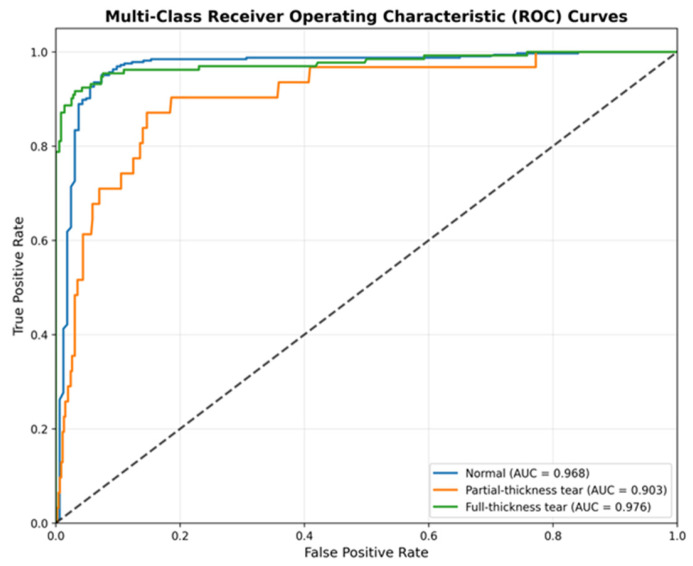
Multi-Class Receiver Operating Characteristic (ROC) Curves. The Area Under the Curve (AUC) indicates robust separability: Normal (0.968), Partial-Thickness (0.903), and Full-Thickness (0.976). The grey dashed diagonal line denotes the chance-level performance of a random classifier (AUC = 0.5).

We further quantified the discriminative power of the model using One-vs-Rest Receiver Operating Characteristic (ROC) curves (Figure 4). This analysis assesses the model’s performance independent of specific decision thresholds.

The ROC analysis corroborates the robustness of the classifier. The model achieves an AUC of 0.976 for the “Full-Thickness Tear” class and 0.968 for the “Normal” class, indicating near-perfect separability. Most notably, for the minority “Partial-Thickness Tear” class, the model achieves an AUC of 0.903. This high AUC value, despite the moderate accuracy observed in the confusion matrix, implies that the model consistently assigns higher probability scores to partial tears compared to negatives. This suggests that the sensitivity for partial tears could be further optimized in a clinical setting by calibrating the decision threshold specifically for this class.

### 3.3. Explainable AI for Clinical Validation

A critical barrier to the clinical adoption of deep-learning systems is their perceived “black box” nature. To validate that the Pa-ViT model relies on genuine anatomical features rather than spurious correlations or artifacts, we utilized Attention Rollout to visualize the self-attention maps generated by the final Transformer block. These heatmaps represent the regions of the MRI slice that most influenced the model’s decision.

**Figure 5:**
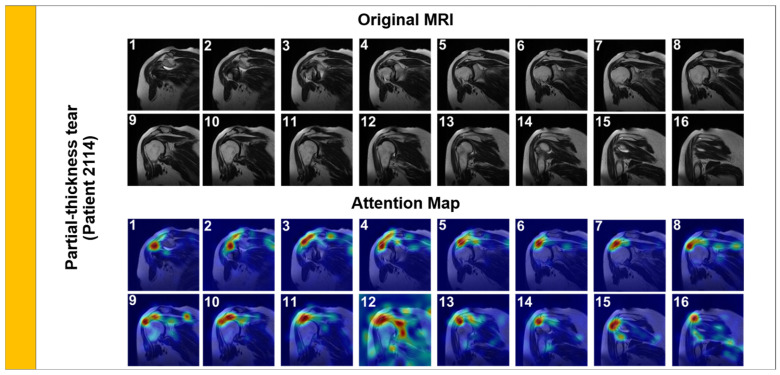
Attention Rollout visualization for a Partial-Thickness Tear (Patient 2114). The overlaid heatmaps (red/yellow regions) demonstrate that the model’s attention is tightly focused on the articular side of the supraspinatus tendon footprint.

We first analyzed a Partial-Thickness Tear (Figure 5, Patient 2114). Radiologically, these tears are characterized by focal signal hyperintensity on the articular or bursal side of the tendon, without complete disruption of the fibers.

As shown in Figure 5, the model’s attention is tightly focused on the articular side of the supraspinatus tendon footprint. This precise localization confirms that the model is detecting the subtle ‘footprint’ lesion rather than general joint inflammation, aligning with expert radiological assessment.

**Figure 6:**
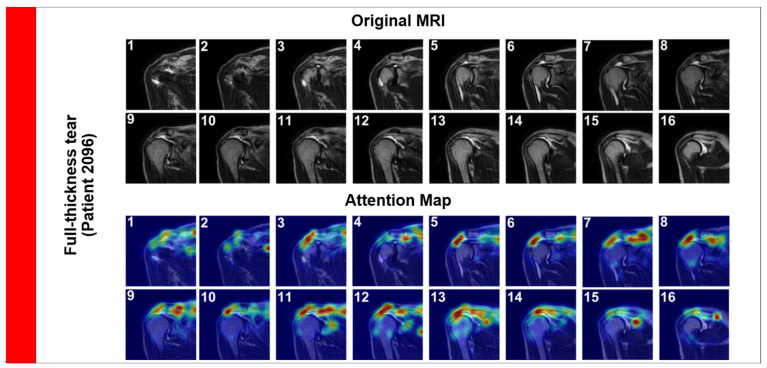
Attention Rollout visualization for a Full-Thickness Tear (Patient 2096). The attention map expands significantly to cover the gap created by the retracted tendon.

Next, we examined a Full-Thickness Tear (Figure 6, Patient 2096). These are morphologically distinct, involving the complete detachment of the tendon from the humeral head, often accompanied by retraction and fluid filling the gap.

In this case, the attention map expands significantly to cover the gap created by the retracted tendon. The model accurately tracks the fluid signal (hyperintensity) filling the space between the humeral head and the acromion. This effectively replicates the ‘fluid sign’ used by radiologists to diagnose full tears, demonstrating the model’s ability to identify complex pathological signs.

**Figure 7:**
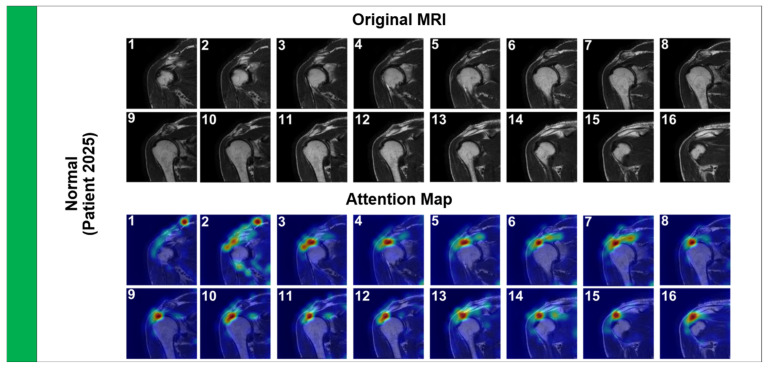
Attention Rollout visualization for a Normal subject (Patient 2025). The attention pattern is diffuse and spreads along the entire length of the intact tendon-bone interface.

Finally, we analyzed a Normal case (Figure 7, Patient 2025) to ensure the model does not produce false positive activations on healthy anatomy.

The attention pattern here is markedly different: it is diffuse and spreads along the entire length of the intact tendon–bone interface. This diffuse attention suggests that the model is verifying the structural continuity of the tendon rather than focusing on a focal pathology. Across representative cases, attention rollout consistently emphasizes clinically meaningful regions: for normal examinations, attention remains distributed along an intact supraspinatus tendon without a focal discontinuity; for partial-thickness tears, attention concentrates on focal footprint/substance signal alterations extending across multiple adjacent slices; and for full-thickness tears, attention peaks around the tendon discontinuity and the fluid-filled gap/retraction pattern. Importantly, because the final prediction is produced after slice-to-patient aggregation (patient-level MIL), the decision is driven by consistent evidence across a range of slices rather than a single isolated slice. This qualitative behavior helps mitigate the “black-box” concern by demonstrating that the model’s discriminative cues align with expected tear morphology. Collectively, these visual explanations provide strong qualitative evidence that the Pa-ViT model’s high quantitative performance is underpinned by a robust, medically grounded understanding of shoulder anatomy and pathology.

## 4. Discussion

This study introduces the Patient-Aware Vision Transformer (Pa-ViT), a novel deep-learning framework that fundamentally redefines the automated diagnosis of Rotator Cuff Tears (RCT) by shifting the computational paradigm from local, slice-level convolution to global, patient-level attention. By achieving a high accuracy of 91% and a macro-averaged F1-score of 0.91 on a challenging, highly imbalanced dataset, our model demonstrates that the inductive biases of Transformers—specifically their ability to model long-range dependencies via Multi-Head Self-Attention—are inherently superior to traditional Convolutional Neural Networks (CNNs) for volumetric medical imaging tasks [4,15,27].

The most significant finding of our work is the substantial performance margin between our Pa-ViT model and the baseline VGG-16 model proposed by Kim et al. [26], which achieved 87% accuracy using a weighted linear combination of 2D slices. While CNN architectures like VGG-16, ResNet, or DenseNet effectively detect local textures, they struggle to contextualize features across large spatial distances. This capability is critical for RCT diagnosis, where the pathological signal requires understanding the geometric relationship between the supraspinatus tendon footprint, the humeral head, and the acromion. Furthermore, recent studies employing 3D CNNs, such as the Voxception-ResNet by Shim et al. (92.5% accuracy) [22] and multi-input CNNs by Yao et al. (AUC 0.94) [20], have attempted to capture this volumetric context. However, these 3D architectures are often computationally prohibitive and data-hungry. Our Pa-ViT model offers a competitive alternative by leveraging the efficiency of 2D slice processing while achieving 3D-like global understanding through the Transformer’s attention mechanism, mirroring the “global processing” cognitive model utilized by expert radiologists.

A critical advancement of this framework lies in bridging the diagnostic gap for Partial-Thickness Tears, which represent the most challenging category in automated shoulder diagnosis due to their subtle radiological presentation and high inter-observer variability. Previous attempts using CNNs have yielded suboptimal results for this class; notably, the baseline study by Kim et al. [26] reported a classification accuracy of approximately 38% for partial tears, with a Precision-Recall area of only 0.45. Similarly, Yao et al. [20] reported a sensitivity of only 72.5% for partial-thickness tears compared to 100% for full-thickness tears, highlighting this class as a universal bottleneck in AI diagnostics. In contrast, our Pa-ViT framework achieved a classification accuracy of 51.61% and an ROC AUC of 0.903 for this minority class. This substantial improvement is attributable to the synergy between our Weighted Cross-Entropy Loss, which effectively counteracted the extreme class imbalance (6.4% prevalence), and the high spatial fidelity of the ViT patch embeddings. These embeddings preserve fine-grained anatomical details often lost during the aggressive pooling operations of deep CNNs. Furthermore, the confusion matrix reveals that the majority of misclassifications for partial tears were “over-estimations” into the Full-Thickness class rather than “under-estimations” into the Normal class. In a clinical decision support context, this bias is preferable as it ensures that patients with potential pathology remain within the pathway for specialist review rather than being falsely cleared, aligning with the conservative management strategies often employed for equivocal cases.

The efficacy of our approach is further underpinned by the formulation of the diagnostic task as a Weakly-Supervised Multiple Instance Learning (MIL) problem with Global Mean Pooling. Rotator cuff tears are inherently volumetric 3D defects [26]; a signal hyperintensity that appears in only a single slice might be an artifact (e.g., magic angle effect), whereas a signal persisting across contiguous slices is likely genuine pathology [16]. The success of our permutation-invariant aggregation strategy confirms that the model successfully learns to filter out slice-specific noise and relies on inter-slice consistency to form a prediction. This renders the Pa-ViT framework robust to stochastic variations in slice thickness or patient positioning—issues that frequently plague 2D slice-based classifiers. Moreover, unlike full 3D segmentation models that require labor-intensive pixel-level annotations [1,28], our MIL approach eliminates the need for dense supervision, making our solution more feasible for deployment in clinical environments with limited hardware resources and annotated data.

Finally, the interpretability provided by our Attention Rollout visualizations addresses the “black box” skepticism often associated with medical AI. Unlike prior CNN-based Class Activation Maps (CAM), which often produce coarse heatmaps, our approach outputs a single patient-level diagnosis (Normal/Partial/Full) with calibrated class probabilities from routinely acquired coronal T2 MRI, without requiring manual ROI annotation or slice-level labeling. This supports use as a triage/second-reader tool: the model can flag suspicious studies, provide class probabilities, and highlight the most influential slices/regions for rapid review, potentially reducing oversight in non-specialist settings. Our visualizations also demonstrate precise anatomical reasoning—identifying the articular footprint in partial tears, tracking the fluid-filled gap in full-thickness tears, and verifying tendon continuity in normal cases. This aligns with the findings of Fazal Gafoor et al. [16], who emphasized the strong correlation between MRI findings and arthroscopy, suggesting that our attention maps could serve as a reliable “second reader” to highlight subtle lesions that might be overlooked in a busy clinical workflow.

Our current patient-level aggregation is permutation-invariant (mean pooling), which is simple and robust to variable-length inputs and heterogeneous acquisition protocols. However, explicitly modeling sequential structure or 3D continuity across slices may further improve sensitivity to subtle partial-thickness tears. Although our aggregation is order-invariant, consistent evidence across multiple adjacent slices can still influence the final patient-level prediction through slice-to-patient pooling, as also supported by our attention rollout analysis. As future work, we plan to investigate sequential aggregation strategies (e.g., attention pooling or a Transformer/RNN over slice embeddings with positional encoding) to better exploit inter-slice relationships while maintaining patient-level outputs.

Although the proposed Pa-ViT framework effectively aggregates slice-level representations using a permutation-invariant MIL formulation, it does not explicitly model slice order or sequential dependencies within the MRI stack. Prior studies have shown that sequential modeling—using attention-based MIL or recurrent architectures—can further enhance clinical decision-making by emphasizing diagnostically salient slices [24,29]. Incorporating explicit slice-level sequential modeling and temporal attention mechanisms, therefore, represents a promising direction for future work and will be explored in subsequent studies to further improve clinical utility [25].

Several limitations of our study must be acknowledged. First, the retrospective, single-center design may limit the generalizability of our findings. Second, the model’s performance was not compared with arthroscopic or open surgical findings, which remain the definitive gold standard for rotator cuff assessment; future studies should incorporate surgical correlation to further validate diagnostic accuracy. Additionally, our current mean pooling method treats slices as an unordered set, potentially overlooking spatial continuity along the z-axis. Lastly, the inherent subjectivity in distinguishing high-grade partial tears from small full-thickness tears remains a challenge, underscoring the need for external validation across multicenter datasets with varying field strengths (1.5T and 3.0T).

## 5. Conclusions

In this study, we introduced the Patient-Aware Vision Transformer (Pa-ViT), a unified framework designed to overcome the limitations of traditional CNN-based architectures in the volumetric diagnosis of rotator cuff tears. By integrating a Vision Transformer backbone with a Weakly-Supervised Multiple Instance Learning paradigm, our approach successfully captures global anatomical context and effectively aggregates volumetric data, addressing critical gaps in automated shoulder MRI analysis.

The experimental validation on a large-scale dataset demonstrated that Pa-ViT achieves strong performance with an accuracy of 91% and an F1-score of 0.91, significantly outperforming conventional baselines. Clinically, the most impactful contribution of this framework is its superior sensitivity in detecting partial-thickness tears, a diagnosis that is historically challenging for both automated systems and varying levels of radiological expertise. Furthermore, the generation of precise, anatomically aligned attention maps bridges the gap between “black-box” deep learning and clinical trust, confirming that the model relies on genuine pathological features rather than spurious correlations.

Ultimately, this work provides a methodological template for future research in medical image analysis, proving the efficacy of self-attention mechanisms over standard convolutions for musculoskeletal pathology. Future directions will focus on enhancing the framework’s clinical utility by integrating multimodal data—such as patient history and demographics—and extending the architecture to multitask learning for simultaneous tear detection and segmentation.

In conclusion, the Pa-ViT framework represents a significant step forward in the automated diagnosis of rotator cuff pathology, offering a clinically viable, accurate, and interpretable solution that outperforms traditional CNN baselines, particularly in the challenging diagnosis of partial-thickness tears.

## Figures and Tables

**Table 1:** Statistical distribution of the shoulder MRI dataset across Training and Validation/Testing subsets. The data partitioning was performed at the patient level to strictly prevent data leakage.

Statistics	Training	Validation/Testing
Total number of examinations (%)	1959 (100)	488 (100)
- Normal examinations (%)	1303 (66.51)	325 (66.60)
- Partial-thickness tear examinations (%)	126 (6.43)	31 (6.35)
- Full-thickness tear examinations (%)	530 (27.06)	132 (27.05)

**Table 2:** Comprehensive performance comparison of the proposed ViT-based method against baseline approaches.

Model	Accuracy	Precision	Recall	F1 Score
Logistic Regression	0.72	0.67	0.72	0.67
AdaBoost	0.66	0.44	0.66	0.53
K-Nearest Neighbors	0.67	0.61	0.67	0.63
Decision Tree	0.68	0.63	0.68	0.65
Random Forest	0.73	0.72	0.73	0.66
Multi-layer Perceptron	0.71	0.66	0.71	0.66
Gaussian NB	0.52	0.67	0.52	0.56
Quadratic Discriminant Analysis	0.57	0.55	0.57	0.56
Gaussian Process	0.61	0.60	0.61	0.60
XGBoost	0.74	0.69	0.74	0.69
Convolutional Neural Networks [26]	0.87	0.81	0.87	0.84
Proposed Method	0.91	0.92	0.91	0.91

## Data Availability

The dataset used in this study is based on the publicly released shoulder MRI dataset introduced by Kim et al. (2447 examinations; coronal T2-weighted slices) [26]. Our complete training and inference code, along with the patient-level evaluation pipeline, will be made available to support reproducibility.
